# Pericoronary Adipose Tissue Attenuation in Patients with Spontaneous Coronary Artery Dissection According to Emotional Versus Physical Triggers: An Analysis from the INSIGHT-SCAD Study

**DOI:** 10.3390/jcdd13050192

**Published:** 2026-04-30

**Authors:** Filippo Luca Gurgoglione, Laura Torlai Triglia, Gabriella Dallaglio, Rebecca Navacchi, Andrea Caraffini, Benedetta Frassoni, Chiara Martini, Gloria Cicala, Alessandro Palumbo, Mattia De Gregorio, Martina Cancellara, Matteo Dalla Bella, Stefano Vago, Giorgio Benatti, Manjola Noni, Rossella Giacalone, Andrea Denegri, Iacopo Tadonio, Davide Donelli, Luigi Vignali, Massimo De Filippo, Giampaolo Niccoli, Emilia Solinas

**Affiliations:** 1 Division of Cardiology, Parma University Hospital, 43126 Parma, Italy; filippolucagurgoglione@gmail.com (F.L.G.); laura.torlaitriglia@unipr.it (L.T.T.); gabriella.dallaglio@unipr.it (G.D.); rebecca.navacchi@unipr.it (R.N.); andrea.caraffini@unipr.it (A.C.); benedetta.frassoni@unipr.it (B.F.); mattia.de.gregorio@gmail.com (M.D.G.); martina.cancellara@unipr.it (M.C.); matteo.dallabella@unipr.it (M.D.B.); stefano.vago@unipr.it (S.V.); gbenatti@ao.pr.it (G.B.); mnoni@ao.pr.it (M.N.); rgiacalone@ao.pr.it (R.G.); adenegri@ao.pr.it (A.D.); itadonio@ao.pr.it (I.T.); luigi.vignali@unipr.it (L.V.); 2 Fondazione Ricerca e Innovazione Cardiovascolare, 20143 Milan, Italy; 3 Division of Radiology, Parma University Hospital, 43126 Parma, Italygcicala@ao.pr.it (G.C.); alepalumbo@gmail.com (A.P.); massimo.defilippo@unipr.it (M.D.F.); 4 Cardiology Unit, Ercole Franchini Hospital, AUSL-IRCCS Reggio Emilia, 42027 Montecchio Emilia, Italy; donelli.davide@gmail.com

**Keywords:** spontaneous coronary artery dissection, pericoronary adipose tissue (PCAT), fat attenuation index (FAI), emotional stress trigger, physical stress trigger, coronary inflammation, non-traditional cardiovascular risk factors, vascular inflammation imaging

## Abstract

Background: the pathophysiological mechanisms underlying spontaneous coronary artery dissection (SCAD) remain incompletely understood. Inflammation may play a pivotal role by promoting vascular susceptibility to SCAD. This study aimed to evaluate pericoronary adipose tissue (PCAT) attenuation, a recognized imaging marker of vascular inflammation, in patients with SCAD. Methods: patients with SCAD who underwent coronary computed tomography angiography (CCTA) within 48 h of the index event and with an identifiable trigger were included. Patients were classified according to the trigger preceding the event (emotional vs. physical). PCAT attenuation was measured in culprit and non-culprit vessels in all patients. Results: A total of 25 SCAD patients were included (mean age 55 ± 11 years, 80.0% female). Emotional triggers were reported in 17 patients (68.0%), while 8 (32.0%) experienced a physical trigger. Type 2 dissections were more common in the emotional trigger group (64.7% vs. 25.0%, *p* = 0.040). Patients with emotional triggers exhibited higher PCAT attenuation compared with those with physical triggers in the SCAD-related vessel (−62.35 ± 6.46 HU vs. −70.86 ± 8.45 HU; *p* = 0.028) and in non-culprit vessels (−61.39 ± 7.24 HU vs. −71.16 ± 5.28 HU; *p* = 0.001). Conclusions: patients with SCAD demonstrated elevated PCAT attenuation, particularly in those with emotional triggers, in both culprit and non-culprit vessels. These findings suggest that vascular inflammation may represent a predisposing factor for SCAD and a target for preventive and therapeutic strategies.

## 1. Introduction

Spontaneous coronary artery dissection (SCAD) is an underrecognized important cause of acute coronary syndrome (ACS), predominantly affecting middle-aged women [1]. Its pathophysiology is multifactorial and reflects a complex interplay between predisposing conditions, including genetic, hormonal, inflammatory, vascular, and psychosocial factors, and precipitating stressors, such as emotional and physical triggers [2].

Identifying the precipitating trigger carries important clinical implications [3,4]. Emotional triggers have been associated with a distinct SCAD phenotype characterized by a lower prevalence of traditional cardiovascular risk factors, a higher burden of psychosocial stress and systemic inflammation, and an increased risk of recurrent angina during mid-term follow-up [5,6].

Coronary computed tomography angiography (CCTA) has emerged as a valuable imaging modality for both diagnosis and longitudinal follow-up of SCAD, providing a noninvasive means of assessing vessel healing and remodeling while avoiding the risks associated with repeat invasive coronary angiography [7]. Notably, CCTA also enables quantification of pericoronary adipose tissue (PCAT) attenuation, measured by the perivascular fat attenuation index (pFAI), a validated imaging marker of coronary inflammation [8].

Recent evidence has shown that patients with recent SCAD exhibit increased coronary inflammatory activity, as demonstrated by higher PCAT attenuation on CCTA in both SCAD-related and non-culprit vessels compared with the general population [9]. Whether PCAT attenuation varies according to the nature of the precipitating stressor, however, remains unknown.

The aim of this study was to investigate the association between the type of precipitating stressor and CCTA-derived PCAT attenuation, assessing both culprit and non-culprit vessels in patients with recent SCAD.

## 2. Materials and Methods

### 2.1. Study Design and Population

This was a single-center mixed retrospective-prospective observational study including consecutive patients who underwent coronary angiography for suspected ACS and with angiographic evidence of SCAD at the University Hospital of Parma between January 2013 and June 2025. The study flow chart is summarized in Supplementary Figure S1.

For the present analysis, we included only patients with SCAD who underwent CCTA within 48 h of the index event and for whom a clear precipitating trigger could be identified. Precipitating stressors were categorized as follows:

- Emotional trigger: significant emotional distress within 24 h before the ACS event, defined as being strongly upset, tense, anxious, worried, or experiencing other forms of extreme or unusual emotional distress.
- Physical trigger: extreme or unusual physical exertion within 24 h before the ACS event [5,6].

Patients were stratified into two study groups according to the nature of the precipitating trigger: emotional versus physical. All patients underwent coronary angiography for suspected ACS and CCTA within 48 h from the acute coronary event. Angiographic characteristics and CCTA variables, particularly PCAT attenuation, were analyzed in both culprit and non-culprit vessels and compared between the two groups.

The study protocol complied with the Declaration of Helsinki, was approved by the institutional Ethics Committee, and all patients provided written informed consent.

### 2.2. Angiographic Analysis

Coronary angiography was performed using radial or femoral access, with multiple projections to visualize all coronary segments. SCAD was angiographically defined as medial dissection or intramural hematoma in the absence of atherosclerosis and classified according to modified version of the Yip and Saw classification (including type 4 SCAD) [10,11], as follows: type 1, pathognomonic contrast dye staining in the arterial wall with multiple radiolucent lumens; type 2, diffuse (typically >20 mm) and usually smooth stenosis, often presenting with abrupt narrowing; type 3, focal or tubular stenosis mimicking atherosclerosis; type 4: abrupt total occlusion. All angiograms were reviewed by two experienced interventional cardiologists (E.S. and L.V.) to confirm the diagnosis.

### 2.3. CCTA Analysis: Methods

The CCTA was performed using a dual-source CT system (Somatom Definition FLASH; Siemens Healthcare, Forchheim, Germany) with specified acquisition parameters. Patients exhibiting heart rates exceeding 65 bpm received intravenous metoprolol to mitigate motion artefacts, while all patients were administered sublingual nitrates to enhance coronary artery visualization. The CCTA protocol includes an initial calcium score quantification using the Agatston method [12], followed by coronary angiography with prospective ECG gating or high-pitch “flash spiral” technique, depending on heart rate. A highly iodinated nonionic contrast agent was administered using the bolus tracking technique. CT acquisition parameters were optimized for image quality and dose reduction, including slice collimation, gantry rotation time, pitch, tube voltage, and tube current. Images were reconstructed using advanced modeled iterative reconstruction (SAFIRE) with a cardiac tissue-specific convolution kernel. All CCTA scans were analyzed using an offline workstation software package (Siemens Healthcare Gmbh—syngo.via—VB60A HF06; Siemens Medical Solutions, Forchheim, Germany). Images were reconstructed in the optimal cardiac phase based on heart rate (typically end-diastole at 60% R–R interval or end-systole at 30%). As per the current literature, four primary SCAD-related coronary features were assessed: (a) intramural hematoma defined as discrete thickening within the wall of the vessel (and not within the lumen, consistent with a Type 2 or 3 SCAD); (b) dissection visualized as a linear hypodensity as would be seen with contrast within the arterial wall consistent with Type 1 SCAD; (c) abrupt luminal stenosis (>50% diameter change over a length of 0.5 mm); (d) tapered luminal stenosis (>50% diameter change over a length of 5 mm) [13].

Perivascular fat attenuation index was measured using a dedicated software package (Aquarius Workstation v4.4.13; TeraRecon Inc., Durham, NC, USA). As previously described, a 40-mm proximal segment was analyzed in each major coronary artery: the right coronary artery (RCA) (commencing 10 mm distal to the ostium), and the left anterior descending (LAD) and left circumflex (LCX) arteries (both commencing at the ostium), as previously described [14]. After identifying the segment of interest, the lumen, the inner and outer wall borders were automatically tracked with additional manual optimization. PCAT was defined as the adipose tissue located within a radial distance from the outer vessel wall equal to the diameter of the respective vessel [15]. Voxel attenuation histograms were plotted, and the weighted average attenuation of all voxels between −30–−190 HU (thresholds utilized for adipose tissue definition) within the PCAT volume was employed to calculate the pFAI. We determined the pFAI by quantifying the weighted perivascular fat attenuation after adjusting for technical parameters. Specifically, when a 100-kV voltage was utilized instead of 120-kV, the mean HU value was corrected by dividing by 1.11485 [16].

### 2.4. Statistical Analysis

Categorical variables were expressed as frequencies and percentages and compared using the Chi-Square test. Continuous variables were presented as means ± standard deviation and compared using Student *t* or Mann–Whitney U tests as appropriate. Descriptive analyses were performed to compare clinical characteristics between emotional and physical trigger groups. PCAT analyses were conducted separately for SCAD-related vessels and non-culprit vessels; the latter was defined as the mean pFAI of the major coronary vessels not affected by SCAD.

Pearson’s correlation coefficient (r) and corresponding 95% confidence intervals were calculated using a point–biserial correlation approach to examine the association between trigger type (emotional vs. physical) and pFAI in both SCAD-related and non-culprit vessels.

All tests were two-sided, and a *p* value < 0.05 was considered statistically significant.

Analyses were performed using R (RStudio v2022.07.2).

## 3. Results

### 3.1. Baseline and Angiographic Features

A total of 25 patients with SCAD were included in the analysis (mean age 55 ± 11 years; 80.0% female). An emotional trigger was identified in 17 patients (68.0%), whereas 8 patients (32.0%) experienced a physical trigger.

Non-traditional cardiovascular risk factors were numerically more frequent among patients with an emotional trigger (58.8% vs. 37.5%, *p* = 0.411), although this difference was not statistically significant. In contrast, obesity (62.5% vs. 5.9%; *p* = 0.010) and hyperglycemia (101.73 ± 19.92 vs. 154.33 ± 43.93, *p* = 0.031) were significantly higher and more prevalent in the physical-trigger group. In the emotional-trigger group eosinophils count was significantly higher (0.11 ± 0.08 vs. 0.04 ± 0.04, *p* = 0.032) All other baseline clinical characteristics were comparable between groups (Table 1).

The left anterior descending artery was the most frequent involved vessel (56.0%), and the Yip–Saw type 2 pattern was the most common angiographic phenotype (52.0%). Notably, patients with an emotional trigger exhibited a significantly higher prevalence of type 2 SCAD compared with those with a physical trigger [11 (64.7%) vs. 2 (25.0%); *p* = 0.040]. Detailed angiographic features are summarized in Table 2.

### 3.2. CCTA Analysis: Main Results

No significant differences were observed between patients presenting with an emotional trigger and those with a physical trigger with respect to coronary morphological characteristics. However, patients with physical triggers exhibited a numerically higher prevalence of double-lumen appearance (34.0% vs. 11.8%, *p* = 0.283), tapered vessel configuration (68.0% vs. 41.2%, *p* = 0.202), and parietal wall thickening (50.0% vs. 35.3%, *p* = 0.667), whereas sudden vessel occlusion was numerically more frequent among patients with emotional triggers (29.4% vs. 12.5%, *p* = 0.624) (Table 3).

Patients with emotional triggers consistently demonstrated higher pFAI values compared with those with physical triggers. Vessel-specific analyses showed significantly higher pFAI in the LAD (−59.69 ± 7.79 HU vs. −70.00 ± 9.71 HU; *p* = 0.023) and RCA (−66.72 ± 6.17 HU vs. −75.36 ± 6.03 HU; *p* = 0.005), with a trend toward higher values in the LCX (−58.73 ± 9.88 HU vs. −67.81 ± 10.13 HU; *p* = 0.054).

**Figure 1:**
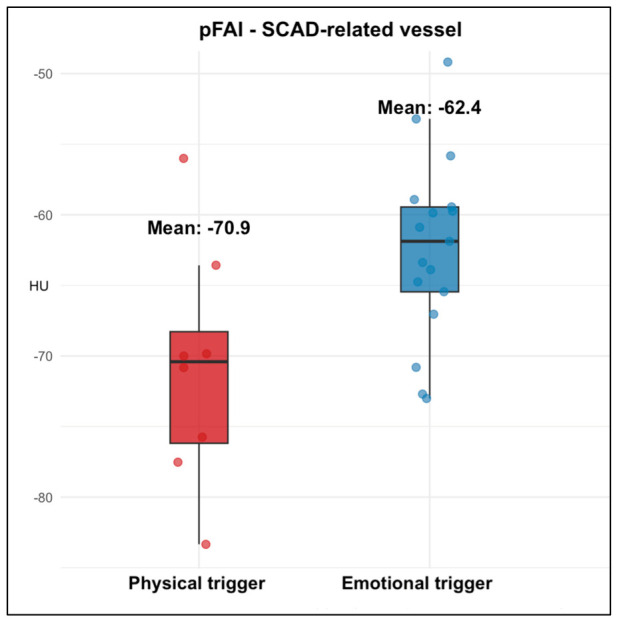
Box plot showing perivascular fat attenuation in SCAD-related vessels according to trigger type.

**Figure 2:**
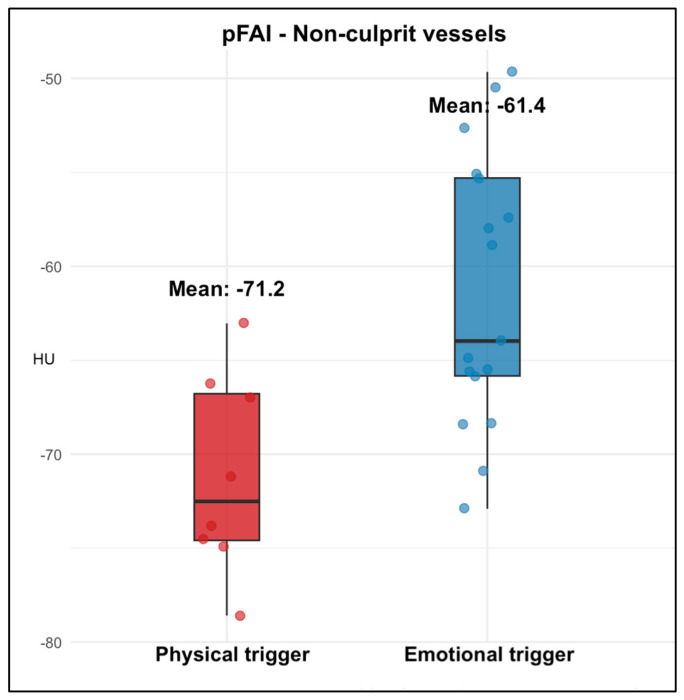
Box plot showing perivascular fat attenuation in non-culprit vessels according to trigger type.

When stratified according to the site of SCAD, patients with emotional triggers exhibited higher pFAI both in the SCAD-related vessel (−62.35 ± 6.46 HU vs. −70.86 ± 8.45 HU; *p* = 0.028) and in non-culprit vessels (−61.39 ± 7.24 HU vs. −71.16 ± 5.28 HU; *p* = 0.001) (Table 3) (Figure 1 and Figure 2).

Finally, correlation analyses demonstrated a significant positive association between emotional triggers and higher pFAI in both SCAD-related and non-culprit vessels. Emotional triggers were moderately correlated with pFAI in the culprit vessel (r = 0.50, 95% CI 0.13–0.75; *p* = 0.010) and strongly correlated with pFAI in non-culprit vessels (r = 0.58, 95% CI 0.24–0.79; *p* = 0.002).

Mean perivascular fat attenuation index (pFAI) values in the culprit vessel among patients presenting with emotional or physical precipitating stressors. Patients with emotional triggers showed significantly higher pFAI.

Comparison of pFAI values in non-culprit coronary vessels according to the type of trigger. Emotional triggers were associated with markedly higher pFAI in non-culprit arteries.

## 4. Discussion

**Figure 3:**
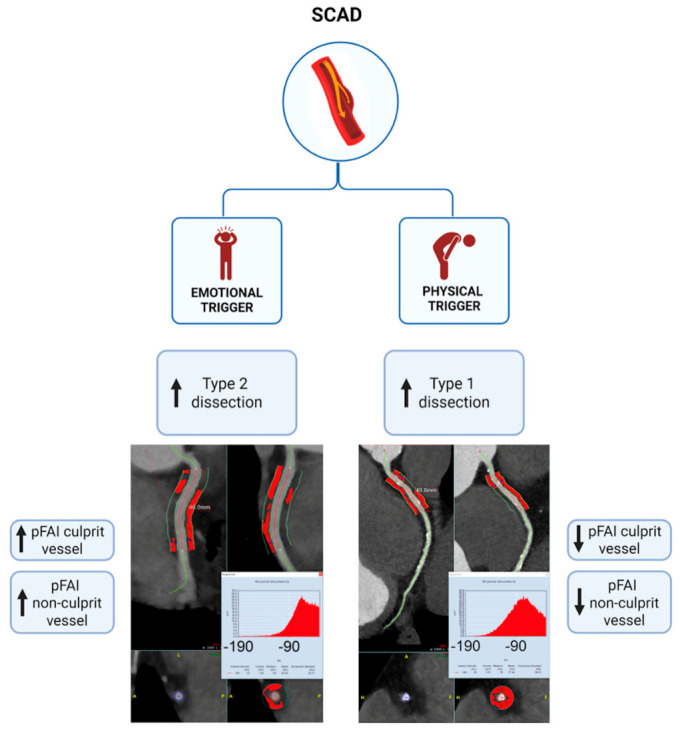
**Central illustration.** Perivascular fat attenuation in SCAD according to trigger type. Summary of the main results of the study. SCAD, spontaneous coronary artery dissection. Created in https://BioRender.com (accessed on 26 February 2026).

In our study, patients presenting with an emotional trigger preceding the SCAD event exhibited higher PCAT attenuation on CCTA performed within 48 h of presentation compared with those experiencing a physical trigger (Central Illustration, Figure 3). PCAT attenuation on CCTA, as reflected by lower pFAI values, has emerged as a validated imaging marker of vascular inflammation. Inflammatory activation alters the metabolic and structural properties of perivascular adipocytes, leading to smaller cells with reduced lipid content, resulting in higher attenuation on CCTA [14]. Clinically, this is highly relevant, as elevated PCAT attenuation has been consistently associated with macrophage-rich coronary plaques on optical coherence tomography [17], increased coronary 18F-sodium fluoride uptake on positron emission tomography [18] and has been described in patients with myocardial infarction with non-obstructive coronary arteries [19]. Currently, reference values for normal pFAI in the general population have not been fully established. The CRISP-CT study demonstrated that a pFAI value ≥ −70.1 HU is a reliable predictor of increased cardiac and all-cause mortality, independent of traditional cardiovascular risk factors [14].

Accumulating evidence has demonstrated clear clinical differences among patients with SCAD according to the nature of the precipitating stressor. Emotional stressors most frequently precede SCAD in women and commonly arise in a background of chronic psychological stress and non-traditional (sex-specific and gender-specific) cardiovascular risk factors [5,6]. In contrast, physical stressors are more often reported in men and are associated with a higher prevalence of traditional cardiovascular risk factors. In a previous study from our group, emotional triggers were associated with higher circulating levels of C-reactive protein and eosinophils, suggesting the hypothesis of a chronic systemic inflammatory state in this SCAD subgroup [6].

The present study extends these observations by demonstrating that patients with emotional triggers have significantly higher levels of PCAT attenuation compared with those experiencing physical triggers. This finding is consistent with the prior, matched analysis by Wolny et al., which showed that patients with recent SCAD have higher PCAT attenuation than age-, sex-, and risk factor-matched controls without SCAD, suggesting heightened vascular inflammation [9]. The association between SCAD and inflammation is increasingly recognized and remains an active area of investigation [20]. The pFAI values observed in our cohorts indicate a high degree of pericoronary fat inflammation and are consistent with existing literature on acute coronary syndromes. Qi et al. recently reported pFAI values in patients with acute myocardial infarction (−76.49 ± 6.67 HU), stable angina (−81.52 ± 6.84 HU), and controls (−84.73 ± 6.35 HU) [21]. In an observational study comparing ACS caused by SCAD (22 patients) with atherosclerotic ACS (30 patients) and 30 healthy controls, SCAD patients demonstrated comparable levels of systemic inflammation compared to atherosclerotic ACS and higher leukocyte and neutrophil counts compared with healthy individuals [22].

Importantly, our data are consistent with the hypothesis that this inflammatory milieu is not limited to the dissected vessel. Patients with emotional triggers exhibited higher PCAT attenuation both in the SCAD-related vessel and non-culprit vessels. Notably, the magnitude of inflammation was comparable between culprit and non-culprit vessels, and the correlation between emotional triggering and PCAT attenuation was even stronger in non-culprit vessels (r = 0.58, *p* = 0.002) than in the SCAD-related artery (r = 0.50, *p* = 0.010). Moreover, the higher eosinophilic count in this group may be consistent with the SCAD eosinophilic infiltration hypothesis and the mechanism of vasa vasorum fragility plus inflammatory adventitial activation [23]. Together, these observations suggest a generalized coronary inflammatory activation in patients with SCAD, particularly in those presenting after an emotional trigger.

From a pathophysiological perspective, the neurohumoral circuits involved in the bidirectional heart–brain interactions and with the stress-inflammation axis, may play a central role in the development of SCAD [24,25,26]. Two distinct patterns of stress have been described. Chronic psychological stress is associated with a chronic low-grade inflammatory state and the release of pro-inflammatory cytokines [27,28,29]. In contrast, acute emotional stress induces a rapid autonomic imbalance with sympathetic overactivation, leading to catecholamine release, enhanced production of pro-inflammatory cytokines, especially IL-1β and IL-6 [30], and endothelial dysfunction [31,32]. In this context, emotional stress may act as a precipitating trigger for SCAD in predisposed individuals, particularly those with an underlying chronic inflammatory milieu [33]. The joint action of heightened vascular inflammation, increased density of vasa vasorum, and recruitment of inflammatory cells into the adventitia of the affected coronary segment may create a vulnerable arterial milieu [23], ultimately facilitating the onset of SCAD in response to an acute emotional stressor.

These findings may also carry important therapeutic implications. Given the elevated levels of systemic inflammatory biomarkers and the diffuse increase in PCAT attenuation observed among patients with emotional triggers, coronary inflammation may represent a promising therapeutic target in selected individuals with SCAD. This perspective may guide the development of targeted preventive strategies, including anti-inflammatory therapies or interventions aimed at reducing oxidative stress and improving endothelial function. This is particularly relevant considering the 10–22% recurrence rate of SCAD [34] and the well-established association between elevated CRP levels after acute coronary events and an increased risk of future cardiovascular complications [35]. Of note, inflammatory activation may also promote oxidative stress, extracellular matrix degradation, and impaired vascular healing, thereby predisposing to new dissections, either in different coronary segments or at the same site [9]. In line with this concept, a recent study evaluating medium- to long-term outcomes in SCAD patients found that a high neutrophil-to-lymphocyte ratio was associated with adverse events, driven primarily by unplanned revascularization [36].

Preliminary studies have also demonstrated reductions in PCAT attenuation among patients with chronic coronary syndrome treated with statins [37] or colchicine [38]. Whether similar anti-inflammatory effects can be achieved in patients with recent SCAD remains unknown and warrants dedicated investigation. Moreover, interventions aimed at reducing psychological stress, through behavioral, psychotherapeutic, or lifestyle-based approaches, may represent an additional therapeutic avenue to mitigate coronary inflammation [39] and potentially reduce the risk of SCAD recurrence.

## 5. Conclusions

In this cohort of patients with recent SCAD, emotional triggers were associated with significantly higher PCAT attenuation on CCTA—both in the SCAD-related artery and in non-culprit vessels—compared with physical triggers. These findings suggest that emotional stress is linked to a more pronounced and diffuse coronary inflammatory activation, supporting the emerging concept of a distinct inflammatory phenotype in emotionally triggered SCAD. The results reinforce the relevance of neuro-immune mechanisms in SCAD pathophysiology and highlight the potential clinical value of recognizing emotional stressors when assessing patient risk profiles and considering future anti-inflammatory or stress-modulating strategies.

## 6. Limitations

This study is limited by its small sample size, single-center design, and reliance on patient-reported triggers, including absence of objective psychological stress measures, which may introduce recall bias. Another important limitation is the absence of non-SCAD control group. PCAT attenuation was assessed at a single time point, preventing evaluation of temporal inflammatory dynamics and it could be possibly the expression of reactive inflammation rather than causative. Although PCAT is a validated marker of vascular inflammation, its reproducibility is still low. Furthermore, inflammatory markers, including CRP, IL-6, hsCRP, and baseline stress levels, were not assessed. Therefore, the findings should be considered exploratory and require confirmation in larger, longitudinal studies integrating imaging and biomarker assessment.

## Figures and Tables

**Table 1:** Baseline clinical features in the overall population and in the two study groups.

Variable	Overall Cohort (n = 25)	Emotional Trigger (n = 17)	Physical Trigger (n = 8)	*p*-Value
Age (mean ± SD)	55 ± 11	57 ± 10	55 ± 11	0.905
Female sex (%)	20 (80.0)	14 (82.4)	6 (75.0)	1.000
Hypertension (%)	11 (44.0)	8 (47.1)	3 (37.5)	0.986
Obesity (%)	6 (24.0)	1 (5.9)	5 (62.5)	0.010
Diabetes (%)	0 (0.0)	0 (0.0)	0 (0.0)	1.000
Hypercholesterolemia (%)	13 (52.0)	9 (52.9)	4 (50.0)	1.000
Current smoking (%)	12 (48.0)	10 (58.8)	2 (25.0)	0.250
Family history of CAD (%)	4 (16.0)	4 (23.5)	0 (0.0)	0.388
Peripheral arterial disease (%)	3 (12.0)	3 (17.6)	0 (0.0)	0.544
Non-traditional risk factors (%)	13 (52.0)	10 (58.8)	3 (37.5)	0.411
Gynaecological disorder (%)	3 (12.0)	2 (11.8)	1 (12.5)	1.000
Hormone therapy (%)	7 (28.0)	5 (29.4)	2 (25.0)	1.000
Endocrine disorder (%)	4 (16.0)	4 (23.5)	0 (0.0)	0.269
Anxiety disorder (%)	4 (16.0)	3 (17.6)	1 (12.5)	1.000
Depressive syndrome (%)	3 (12.0)	3 (17.6)	0 (0.0)	0.544
LVEF (median [Q1–Q3])	55.0 [45.0–59.0]	55.0 [51.0–59.0]	55.0 [48.7–61.2]	0.567
Non-STEMI (%)	14 (56.0)	9 (52.9)	5 (62.5)	0.771
STEMI (%)	11 (44.0)	8 (47.1)	3 (37.5)	1.000
Laboratory (mean ± SD)				
Hb (g/dL)	13.04 ± 2.10	13.13 ± 2.09	12.84 ± 2.27	0.78
PLT (×10^3^/µL)	271 ± 111	242 ± 88	338 ± 133	0.116
WBC (×10^3^/µL)	8.88 ± 3.13	8.41 ± 2.45	10.0 ± 4.36	0.404
Neutrophils (×10^3^/µL)	6.49 ± 3.36	5.77 ± 2.19	8.03 ± 4.98	0.329
Lymphocytes (×10^3^/µL)	2.56 ± 4.01	3.05 ± 4.80	1.52 ± 0.86	0.288
Eosinophils (×10^3^/µL)	0.09 ± 0.07	0.11 ± 0.08	0.04 ± 0.04	0.032
Basophils (×10^3^/µL)	0.03 ± 0.02	0.03 ± 0.02	0.04 ± 0.02	0.652
Monocytes(×10^3^/µL)	0.55 ± 0.19	0.58 ± 0.20	0.49 ± 0.17	0.324
Creatinine (mg/dL)	0.73 ± 0.17	0.69 ± 0.19	0.81 ± 0.07	0.041
c-LDL (mg/dL)	99.28 ± 34.03	100.54 ± 36.90	96.00 ± 28.61	0.788
Glycemia (mg/dL)	120.29 ± 39.02	101.73 ± 19.92	154.33 ± 43.93	0.031
Medications at discharge (%)				
Aspirin	23 (92.0)	16 (94.1)	7 (87.5)	1.000
DAPT	21 (84.0)	14 (82.4)	7 (87.5)	0.783
NOAC	1 (4.0)	0 (0.0)	1 (12.5)	0.381
Statin	20 (80.0)	13 (76.5)	7 (87.5)	1.000
Beta-blocker	23 (92.0)	15 (88.2)	8 (100.0)	1.000

Abbreviations: CAD: coronary artery disease; c-LDL: low-density lipoprotein cholesterol; Hb: hemoglobin; DAPT: dual antiplatelet therapy; LVEF: left ventricular ejection fraction; NOACs: novel oral anticoagulants; PLTs: platelets; SD: standard deviation; STEMI: ST-elevation myocardial infarction; WBCs: white blood counts.

**Table 2:** Angiographic features in the overall population and in the two study groups.

Variable	Overall Cohort (n = 25)	Emotional Trigger (n = 17)	Physical Trigger (n = 8)	*p*-Value
Involved vessels (%)				0.238
LM	2 (8.0)	2 (11.8)	0 (0.0)	
LAD	14 (56.0)	8 (47.0)	6 (75.0)	
LCX	7 (28.0)	5 (29.4)	2 (25.0)	
RCA	4 (16.0)	4 (23.5)	0 (0.0)	
Multivessel SCAD (%)	2 (8.0)	2 (11.8)	0 (0.0)	1.000
Yip–Saw Classification (%)				0.040
Type 1	7 (28.0)	3 (17.6)	4 (50.0)	
Type 2	13 (52.0)	11 (64.7)	2 (25.0)	
Type 3	1 (4.0)	0 (0.0)	1 (12.5)	
Type 4	4 (16.0)	3 (17.6)	1 (12.5)	
TIMI flow < 3 (%)	3 (12.0)	3 (17.6)	0 (0.0)	0.258

Abbreviations: LAD: left anterior descending; LCX: left circumflex; LM: left main; RCA: right coronary artery; SCAD: spontaneous coronary artery dissection; TIMI: Thrombolysis in Myocardial Infarction.

**Table 3:** CCTA features in the overall population and in the two study groups.

Variable	Overall Cohort (n = 25)	Emotional Trigger (n = 17)	Physical Trigger (n = 8)	*p*-Value
Double-lumen appearance (%)	5 (20.0)	2 (11.8)	3 (34.0)	0.283
Sudden vessel occlusion (%)	6 (24.0)	5 (29.4)	1 (12.5)	0.624
Tapered vessel appearance (%)	13 (52.0)	7 (41.2)	6 (68.0)	0.202
Parietal wall thickening (%)	10 (40.0)	6 (35.3)	4 (50.0)	0.667
pFAI (HU), mean ± SD				
LAD	−62.99 ± 9.59	−59.69 ± 7.79	−70.00 ± 9.71	0.023
RCA	−69.49 ± 7.27	−66.72 ± 6.17	−75.36 ± 6.03	0.005
LCX	−61.63 ± 10.66	−58.73 ± 9.88	−67.81 ± 10.13	0.054
SCAD-related vessel	−65.07 ± 8.06	−62.35 ± 6.46	−70.86 ± 8.45	0.028
Non-culprit vessels	−64.52 ± 8.04	−61.39 ± 7.24	−71.16 ± 5.28	0.001

Abbreviations: HU: Hounsfield unit; LAD: left anterior descending; LCX: left circumflex; RCA: right coronary artery; pFAI: perivascular fat attenuation index; SCAD: spontaneous coronary artery dissection; SD: standard deviation.

## Data Availability

The original contributions presented in this study are included in the article/Supplementary Materials. Further inquiries can be directed to the corresponding authors.

## Supplementary Materials

The following supporting information can be downloaded at: https://www.mdpi.com/article/10.3390/jcdd13050192/s1, Figure S1: Study Flow Chart.

## Abbreviations

The following abbreviations are used in this manuscript:

SCAD	Spontaneous Coronary Artery Dissection
ACS	Acute Coronary Syndrome
CCTA	Coronary Computed Tomography Angiography
PCAT	Pericoronary Adipose Tissue
HU	Hounsfield Unit
pFAI	Perivascular Fat Attenuation Index
LAD	Left Anterior Descending Artery
LCX	Left Circumflex Artery
RCA	Right Coronary Artery
LM	Left Main Coronary Artery
CAD	Coronary Artery Disease
LVEF	Left Ventricular Ejection Fraction
STEMI	ST-Elevation Myocardial Infarction
TIMI	Thrombolysis in Myocardial Infarction
NTRFs	Non-Traditional Risk Factors
PET	Positron Emission Tomography
MINOCA	Myocardial Infarction with Non-Obstructive Coronary Arteries
