# Supplementary figures and images for: Pericoronary Adipose Tissue Attenuation in Patients with Spontaneous Coronary Artery Dissection According to Emotional Versus Physical Triggers: An Analysis from the INSIGHT-SCAD Study
Source: J Cardiovasc Dev Dis. 2026 Apr 30;13(5):192. doi: 10.3390/jcdd13050192 (PMC13207271; doi:10.3390/jcdd13050192)

## Supplementary Figure S1. Study Flow Chart.

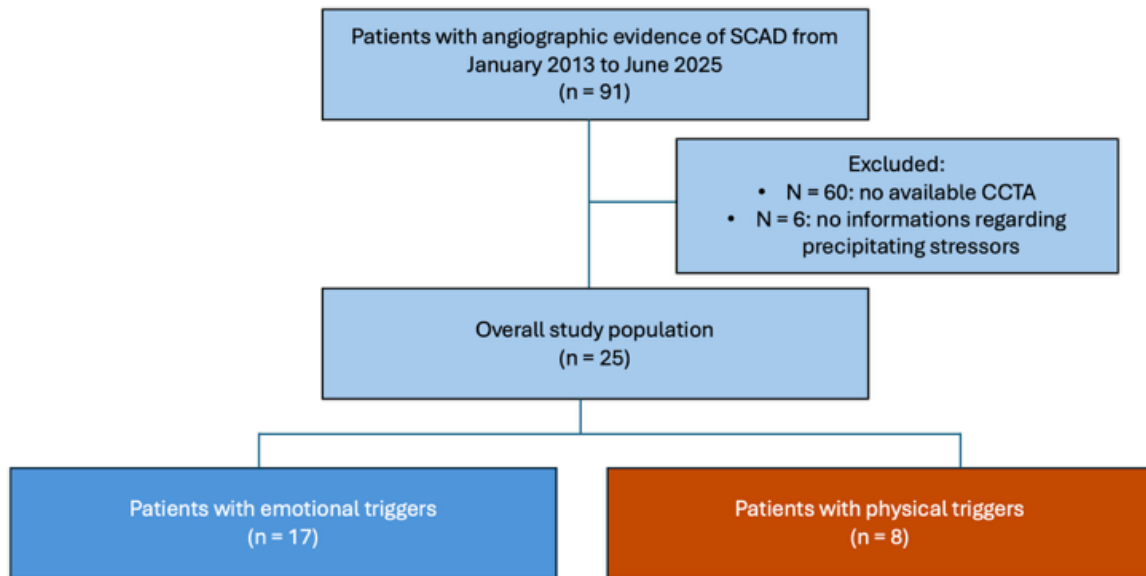

Supplement: Supplementary file 1 [file jcdd-13-00192-s001.zip › jcdd-4211474-supplementary.pdf]
